# Longer Telomere Length in COPD Patients with α_1_-Antitrypsin Deficiency Independent of Lung Function

**DOI:** 10.1371/journal.pone.0095600

**Published:** 2014-04-24

**Authors:** Aabida Saferali, Jee Lee, Don D. Sin, Farshid N. Rouhani, Mark L. Brantly, Andrew J. Sandford

**Affiliations:** 1 UBC James Hogg Research Centre, St. Paul's Hospital, Vancouver, British Columbia, Canada; 2 Department of Medicine, University of Florida, Gainesville, Florida, United States of America; University of Newcastle, United Kingdom

## Abstract

Oxidative stress is involved in the pathogenesis of airway obstruction in α_1_-antitrypsin deficient patients. This may result in a shortening of telomere length, resulting in cellular senescence. To test whether telomere length differs in α_1_-antitrypsin deficient patients compared with controls, we measured telomere length in DNA from peripheral blood cells of 217 α_1_-antitrypsin deficient patients and 217 control COPD patients. We also tested for differences in telomere length between DNA from blood and DNA from lung tissue in a subset of 51 controls. We found that telomere length in the blood was significantly longer in α_1_-antitrypsin deficient COPD patients compared with control COPD patients (p = 1×10^−29^). Telomere length was not related to lung function in α_1_-antitrypsin deficient patients (p = 0.3122) or in COPD controls (p = 0.1430). Although mean telomere length was significantly shorter in the blood when compared with the lungs (p = 0.0078), telomere length was correlated between the two tissue types (p = 0.0122). Our results indicate that telomere length is better preserved in α_1_-antitrypsin deficient COPD patients than in non-deficient patients. In addition, measurement of telomere length in the blood may be a suitable surrogate for measurement in the lung.

## Introduction

Chronic obstructive pulmonary disease (COPD) is a complex trait with both genetic and environmental risks factors that is characterized by non-reversible airway obstruction and chronic inflammation. The morphologic manifestations of this disorder include small airway remodeling and emphysema. The predominant environmental risk factor for COPD is cigarette smoking, [1], [2] although other factors such as air pollution [3], [4] and respiratory infections [5] play a role.

The genetic component of COPD has been demonstrated by twin studies of disease status [6] and lung function [7], [8]. The search for the genes responsible for this disorder has involved the investigation of candidate genes [9] as well as genome-wide association studies of COPD [10], [11] and lung function in the general population [12]–[15]. These studies have identified novel genes such as Hedgehog-interacting protein [11]–[13], [15], [16], α-nicotinic acetylcholine receptor [11], [17] and 5-hydroxytryptamine (serotonin) receptor 4 [12], [13], [15].

While several novel susceptibility genes for COPD have been identified in recent years, the underlying mechanisms are largely unknown. In contrast, the association between deficiency of α_1_-antitrypsin and emphysema has been known for several decades [18], [19] and the pathophysiology is understood [20], [21]. α_1_-antitrypsin is a proteinase inhibitor and acute phase reactant, and its major role is the inhibition of neutrophil elastase. α_1_-antitrypsin deficiency is caused by alleles of the *SERPINA1* gene. Severe deficiency of α_1_-antitrypsin is most often caused by homozygosity for the Z allele (Glu342Lys) of *SERPINA1* and is a risk factor for early-onset emphysema, although the clinical manifestations are highly variable [22], [23].

A recent focus of COPD research has been the role of premature aging of the lung and other organs. Emphysema is characterized by reduced cell proliferation [24] and increased markers of cellular senescence [25], including shortened telomeres [26]. COPD patients are at increased risk for cardiovascular disease [27], osteoporosis [28], depression [29], and skin wrinkling [30], all of which have been associated with premature senescence [31]–[33].

Telomeres shorten with each round of cell division and this results in replicative cell senescence. Telomere length is reduced during DNA replication because of the “end replication problem”, i.e., the 5′ end of the lagging strand is unable to be replicated. This loss of telomeric DNA is predicted to be ∼10 base pairs (bp) per cell cycle. However, the observed rate of loss can be higher and in humans has been estimated to be 50–200 bp per division [34], [35]. Oxidative stress is one of the main factors in causing this higher rate of loss [36], [37].

Several studies have examined telomere length in the context of COPD but there is little consistency in the results [26], [38]–[45] and the relationship between telomere length and lung function in α_1_-antitrypsin deficiency has not been previously studied. Oxidative stress plays an important role in the pathogenesis of airway obstruction in α_1_-antitrypsin deficient patients [46]. Furthermore, α_1_-antitrypsin has anti-apoptotic effects [47], [48] and anti-inflammatory effects on cytokine production [49], [50]. Therefore, cell senescence due to reduction in telomere length may be particularly important in patients with α_1_-antitrypsin deficiency. We investigated telomere length in a group of COPD patients with α_1_-antitrypsin deficiency and a group of COPD controls. We also determined whether the length of telomeres in peripheral blood DNA is correlated with that in lung tissue samples in order to test the hypothesis that COPD is a “systemic” disease, and that telomeric shortening in this condition affects both lung and the hematopoietic systems.

## Methods

### Subjects

We studied 217 α_1_-antitrypsin deficient patients and 217 COPD control patients (Table 1). Approval for the project was obtained from the University of British Columbia - Providence Health Care Research Ethics Board (REB H09-02042 and H11-02780). The α_1_-antitrypsin deficient patients were selected from the Alpha-1 Foundation (AATF) DNA and Tissue Bank located at the University of Florida (IRB 659-2002). The COPD controls were selected from the Lung Health Study (LHS), a clinical trial sponsored by the National Heart, Lung and Blood Institute [51]. Participants in the LHS were cigarette smokers between 35–60 years of age with mild to moderate airflow obstruction, defined by a ratio of forced expiratory volume in one second (FEV_1_) to forced vital capacity ≤0.70 and FEV_1_ between 55–90% of predicted. Selected LHS samples were matched to the α_1_-antitrypsin deficient samples for age, gender, ethnicity and pack years. An additional 51 patients were selected from the lung tissue biobank at the James Hogg Research Centre (JHRC). For the JHRC samples, both lung tissue and blood samples were obtained from patients admitted to St. Paul's Hospital who underwent lobar or lung resection surgery for localized lung cancer. The lung tissue samples were taken from a site distant from the tumor. All subjects provided written informed consent.

**Table 1:** Demographic and genotypic characteristics of subjects from the Alpha-1 Foundation DNA and Tissue Bank and the Lung Health Study.

		Alpha-1 Foundation DNA and Tissue Bank	Lung Health Study
**Age** *		53.7±0.45	53.7±0.45
**% Male**		51.1	51.1
**Pack Years** *		22.8±1.28	23.2±0.92
**Average FEV1% predicted** *		40.0±1.68	79.9±0.59
**Genotype**	MM	0	217
	MZ	0	0
	ZZ	217	0

*Mean ± standard error of the mean.

### DNA samples

A sample of peripheral blood DNA was used to measure telomere length in the AATF DNA and Tissue Bank samples. Measurement of telomere length in the LHS samples was performed as previously reported [43]. For the JHRC samples, we measured telomere length in DNA samples from both blood and lung tissue in 51 subjects. DNA was extracted from these tissues using the QIAamp DNA Mini Kit (Qiagen, Mississauga, ON, Canada).

### Measurement of Telomere Length

Telomere length was measured using a previously published qPCR based protocol [43], [52]. Briefly, DNA samples were quantified using the Nanodrop 8000 spectrophotometer (Thermo Scientific, Wilmington, DE, USA). Telomere length measurement was performed in triplicate using 5 ng of DNA. Intra-plate coefficients of variance (CV) were calculated between the replicates, and samples with CV≥5% were excluded from further analysis. Reference DNA samples obtained from the Coriell Institute (Camden, NJ) were assayed as calibrator samples in triplicate on each PCR plate to control for variation between plates. Inter-plate CV for the calibrator sample was calculated to be 16%. 36B4 was used as a reference gene. The primer sequences used were: tel 1: GGTTTTTGAGGGTGAGGGTGAGGGTGAGGGTGAGGGT; tel 2: TCCCGACTATCCCTATCCCTATCCCTATCCCTATCCCTA; 36B4u: CAGCAAGTGGGAAGGTGTAATCC; and 36B4d: CCCATTCTATCATCAACGGGTACAA. Six qPCR reactions (i.e. triplicates of the telomere and reference gene assays) were performed for each individual in 20 µL reactions including 10 µL QuantiTect SYBR Green PCR Master Mix (QIAGEN), and final primer concentrations of tel 1: 270 nM, tel 2: 900 nM, 36B4u: 300 nM, and 36B4d, 500 nM. Reactions were performed on the ViiA 7 Real-Time PCR Instrument (Life Technologies). Cycling conditions for the measurement of telomere length were as follows: 50°C for 2 min, 95°C for 2 min, 40 cycles of 95°C for 15 sec and an annealing temperature of 54°C for 2 min. Cycling conditions for measurement of the 36B4 reference gene were the same except 35 cycles, with an annealing temperature of 58°C for 1 min were used. Telomere length was calculated as a ratio of telomere to 36B4, using Cawthon's formula [52].

### Statistical Analysis

Telomere length measurements were log_10_ transformed to approximate a normal distribution. Student's t-test was used to compare mean telomere length between groups. Multiple linear regression was performed to test for the effect of α_1_-antitrypsin deficiency on telomere length and lung function, with adjustments for significant confounders including age, gender and pack years. JMP software (SAS, Cary, NC, USA) was used for all statistical analyses.

## Results

### Effect of α_1_-antitrypsin deficiency on telomere length

**Figure 1:**
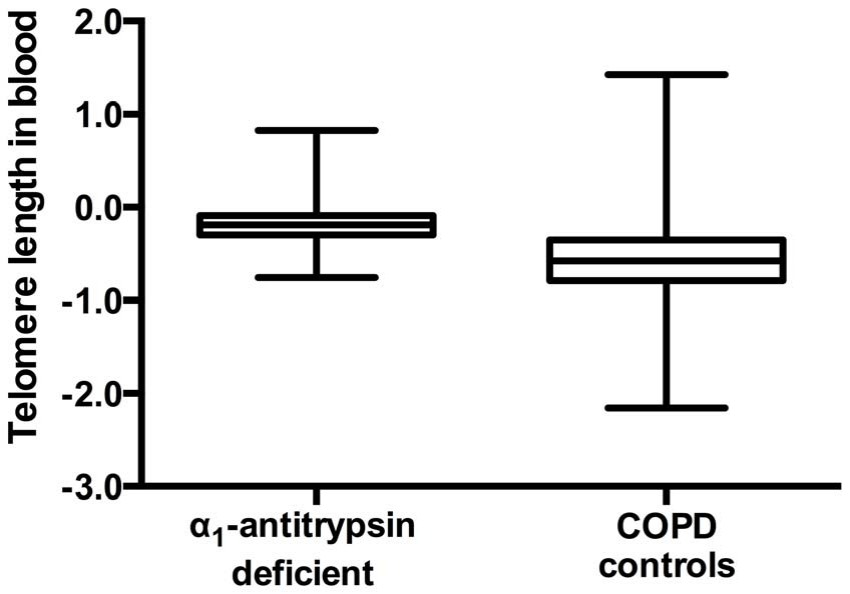
Log_10_ of telomere length in α_1_-antitrypsin deficient COPD patients vs. log_10_ of telomere length in COPD controls.

To test for association between α_1_-antitrypsin deficiency and telomere length in peripheral blood cells, telomere length was measured in 217 α_1_-antitrypsin deficient patients and 217 control patients matched for ethnicity, gender, age and pack years. Mean telomere length was compared between the two groups using Student's t-test. Median telomere length (untransformed) was 2.45 fold longer in peripheral blood DNA from COPD patients with α_1_-antitrypsin deficiency compared with COPD controls (p = 1×10^−29^) (Figure 1). The mean log_10_ transformed telomere lengths in α_1_-antitrypsin deficient patients and COPD controls were −0.1882 with a standard deviation of 0.2074 and −0.5639 with a standard deviation of 0.2074, respectively. The difference in telomere length between α_1_-antitrypsin deficient patients and COPD controls remained statistically significant after adjustment for age, gender and pack years. As a replication cohort, a second set of 217 COPD controls were selected who were matched to the α_1_-antitrypsin deficient patients for ethnicity, gender, age and pack years. Mean telomere length was again significantly longer in the α_1_-antitrypsin deficient patients compared with COPD controls (p = 1×10^−33^).

### Relationship between tissue type and telomere length

**Figure 2:**
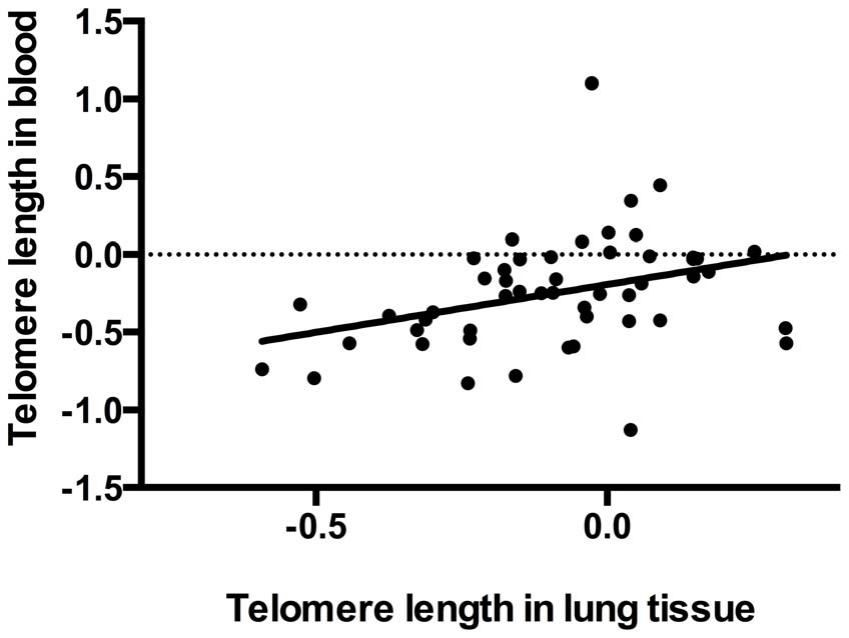
Log_10_ of telomere length in the lung vs. log_10_ of telomere length in the blood.

**Figure 3:**
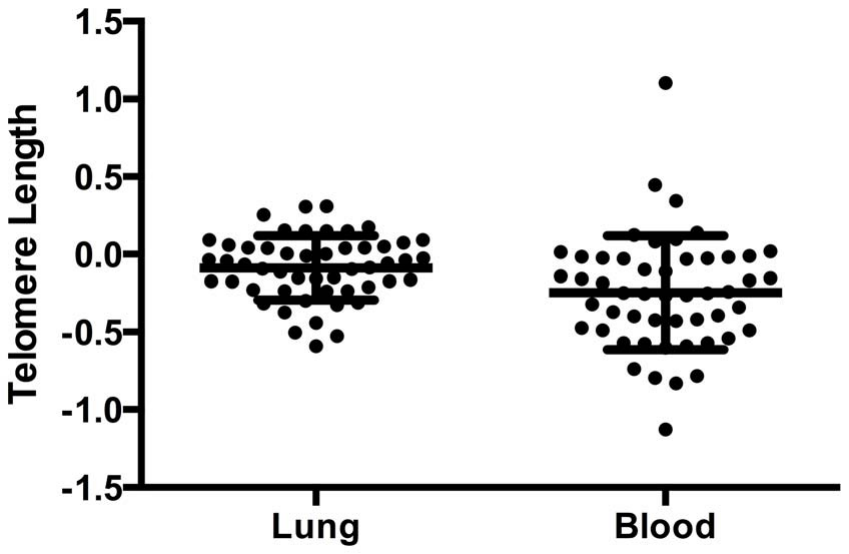
Mean log_10_ of telomere length in the blood and lung tissue.

To test for a correlation between telomere length in the blood and in the lungs, telomere length was measured in lung and blood DNA from 51 patients from the JHRC. There was a significant correlation between telomere length in the blood and telomere length in the lungs (Pearson's r = 0.348, p = 0.012) (Figure 2). On average, however, median telomere length was 1.53 fold shorter in the blood when compared with the lungs (p = 0.008) (Figure 3).

### Relationship between telomere length and lung function

Lung function data were available for 157/217 α_1_-antitrypsin deficient patients and all COPD controls. The effect of telomere length in the blood on FEV_1_ % predicted was tested. There was no significant association between telomere length in the blood and lung function in either the α_1_-antitrypsin deficient patients (p = 0.3122), or in the controls with adjustment for age and pack years (p = 0.2503). In addition, lung function data were available for 49 patients from the JHRC with telomere length measurements in the lung. There was no association between telomere length in the lung and FEV_1_% predicted (p = 0.8057).

## Discussion

The most important finding of this study was that COPD patients with α_1_-antitrypsin deficiency have longer telomere lengths in peripheral leukocytes compared with COPD patients who do not have α_1_-antitrypsin deficiency. However, there was no significant relationship between telomere length in blood and lung function as measured by FEV_1_% predicted in α_1_-antitrypsin deficient patients or in COPD controls. We also found that within subjects, there was a significant relationship of telomere length in peripheral leukocytes with that in lung tissue, although on average the telomere length of peripheral leukocytes was shorter than that in lung tissue.

Telomere length has been positively correlated with lung function in some studies [26], [40], [44], [45] but not others [41], [43]. A recent study examined 46,396 individuals and the results suggested that the association of telomere length with lung function was, though significant, only modest after correction for confounding factors such as age [44]. Shorter telomere length has also been associated with COPD in some studies [39], [41], [42], [44], [45] but not in others [25], [38]. This is the first study to examine the role of telomere length in α_1_-antitrypsin deficient patients, a group who we hypothesized may be particularly susceptible to accelerated reduction in telomeres and the subsequent cellular senescence.

The role of premature aging in COPD has been shown by studies of explanted lung fibroblasts from emphysema patients that showed reduced proliferation rate *in vitro* [24] and markers of cellular senescence [25]. Similarly, alveolar type II cells and endothelial cells from emphysema patients showed elevated levels of senescence markers including shortened telomeres [26].

In our study we found a relationship between α_1_-antitrypsin deficiency and telomere length. However, the direction of effect was contrary to our hypothesis. Telomere length was longer in patients with α_1_-antitrypsin deficiency, despite the fact that they are likely exposed to higher levels of oxidative stress than usual COPD patients, as measured by oxidation of nucleic acids [46]. Oxidation of DNA is a general marker of oxidative stress and may directly promote telomere shortening [53] and therefore our results appear counterintuitive. On the other hand, patients with α_1_-antitrypsin deficiency have lower levels of myeloperoxidase (MPO) and neutrophil counts in sputum than non-deficient COPD patients [54]. MPO is the most abundant protein in neutrophils and catalyzes the formation of hypochlorous acid, a potent oxidant. Therefore, MPO likely plays an important role in oxidative stress in the lung and the lower MPO levels in α_1_-antitrypsin deficient patients [54] may explain the longer telomere length we observed in these patients.

We found that there was a significant correlation between telomere length in the blood and telomere length in lung tissue. Many studies of telomere length are performed using DNA from peripheral blood cells, and results are extrapolated to biological processes occurring in other tissues. For example, the majority of studies investigating telomere length in COPD patients have been performed in DNA from blood cells [24], [38], [45], [47], [48]. Our results indicate that telomere length in the blood is correlated with telomere length in the lungs, suggesting that telomere length in the blood may be an appropriate surrogate for telomere length in the lungs. The correlation between blood and lung telomere length may reflect the nature of COPD as a systemic disease [55]. Thus, exposure to cigarette smoke in the lungs may affect leukocyte telomere length due to translocation of proinflammatory mediators [56] and reactive oxygen species from the lung into the circulation.

We also demonstrated that the telomere length of peripheral leukocytes was shorter than that in lung tissue. Telomere length is known to vary between different human tissues [57] with leukocyte telomeres generally shorter than those in other tissues [58], presumably reflecting greater rates of proliferation in blood cells. Interestingly, Daniali *et al.* [58] studied adults (age >18 years) and the rate of telomere shortening was similar between the tissue types, suggesting that the length differences between tissues were established in childhood. This may explain why the telomeres in the lung samples in our patient samples were longer than those in blood cells, despite the presumably greater exposure of the lung tissue to oxidative stress via cigarette smoke. The telomere length differences established early in life may overwhelm any effect of exposure to smoke occurring mainly in adulthood.

One limitation of our study is that lung function was only measured at one time point; therefore we could not test the effect of telomere length on rate of decline in α_1_-antitrypsin deficient patients. Another limitation is that all of the α_1_-antitrypsin deficient patients included in this study were current or ex-smokers. Therefore, it was not possible to test for the effect of α_1_-antitrypsin deficiency in non-smokers compared with smokers. Finally, our telomere length measurements in the lung were performed using only a small piece of lung tissue, therefore the telomere length measured may not reflect the whole lung.

Our data indicate that in α_1_-antitrypsin deficient patients, replicative senescence does not appear to play a significant role in the pathogenesis of COPD. Importantly, for the respiratory community, we found that telomere length of peripheral leukocytes is a good biomarker of telomere length in lung tissue.
